# Finding an efficient tetramethylated hydroxydiethylene of resveratrol analogue for potential anticancer agent

**DOI:** 10.1186/s13065-020-00667-5

**Published:** 2020-02-18

**Authors:** Zhen-Hui Xin, Ya-Li Meng, Wen-Jing Jiang, Ya-Peng Li, Li-Ping Ge, Cun-Hui Zhang, Lian-Na Liu, Yan-Fei Kang

**Affiliations:** 1 Hebei Key Laboratory of Quality & Safety Analysis-Testing for Agro-Products and Food and College of Laboratory Medicine, Hebei North University, 11 Diamond Street South, Zhangjiakou, 075000 Hebei People’s Republic of China; 2 Zhangbei Hospital, Guangchang Alley, Garden Street, Zhangbei Country, Zhangjiakou, 076450 Hebei People’s Republic of China

**Keywords:** Resveratrol, Cell apoptosis, Cell cycle arrest, ROS

## Abstract

With the improvement and advance in cancer diagnosis and treatment, the cancer is still a major cause of morbidity and mortality throughout the world. Obviously, new breakthroughs in therapies remain be urgent needed. In this work, we designed and synthesized the compound **1**–**4,** namely resveratrol analogues with methylation of hydroxy distyrene, to further explore its new anti-cancer potential. Encouragingly, compound **1** ((*E*)-4,4′-(ethene-1,2-diyl)bis(3,5-dimethylphenol)) exhibited cytotoxicity superior to resveratrol in MCF 7 cells. More importantly, the compound **1** showed greater toxicity to tumor cells than that to normal cells, which proved that it could selectively kill tumor cells. The favorable results encouraged us to explore the inhibitory mechanism of compound **1** on MCF 7 cells. The research finding indicated the compound **1** inhibited tumor cell proliferation by both arresting cell cycle in S phase and apoptosis via a prooxidant manner. In addition, the results further verified compound **1** caused cell cycle arrest in S phase and apoptosis by down-regulation of the cycling A1/cycling A2 expression and the rise of Bax/Bcl-2 ratio in a p21-dependant pathway in MCF 7 cells. Therefore, these results are helpful for the effective design of anticancer reagents and the better understanding of their mechanism of action.

## Introduction

Cancer, as a global public health and social problem, is a major cause of morbidity and mortality, affecting all of humankind [1]. Due to multiple etiologies, complicated pathogenesis, and multi-stages, although great efforts have been made, many kinds of cancers still don’t have efficient screening strategies, or just have limited treatment options with high recurrence risks and/or adverse prognosis. Obviously, there remains an urgent need for effective treatments and new breakthroughs in cancer therapy.

A large body of research has shown that carcinogenesis is associated with the cell cycle control, signal transduction or apoptosis leading to abnormal proliferation of cancer cells, which may provide the potential drug targets for cancer therapies [2–5]. Obviously, molecules with the potential of an anti-proliferation and pro-apoptotic effect on tumor cells, but not on normal cells, may be appropriate anti-cancer agents. Reactive oxygen species (ROS), as the intracellular second messengers, are closely involved in various physiology processes in normal cells. However, once the redox homeostasis is disrupted, ROS will present heterogeneous effects depending on the different levels [6]. ROS may display the carcinogenic effect at low levels through the induction of cellular abnormal proliferation [7]. Interestingly, the basal ROS levels in cancer cells are higher than those in normal cells, which assign the negative role of ROS in tumorigenesis at high levels through prooxidant activity to induce apoptosis and/or cell cycle arrest by damage DNA [8, 9]. Based on this, exploration of prooxidant strategies may be effective therapeutic measures.

**Fig. 1:**
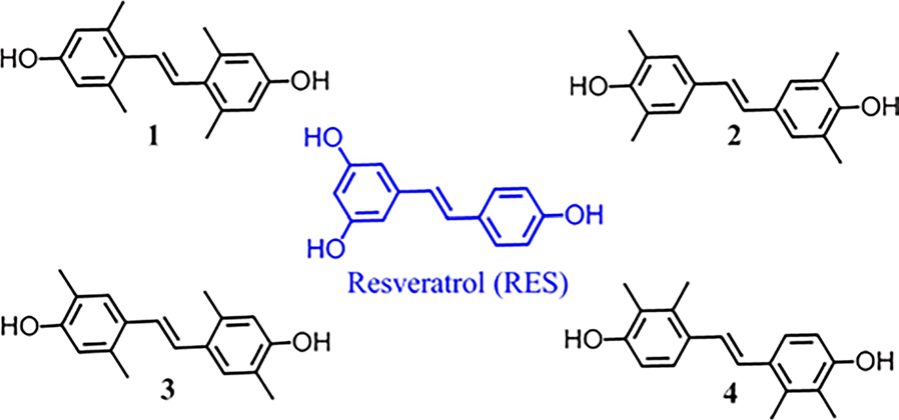
Resveratrol analogues 1–4

Resveratrol (3,4′,5,-trihydroxy-trans-stilbene, RES), as a kind of natural polyphenolic compound in a wide variety of dietary, has attracted a large number of attention. Because of its pharmacological properties, RES has been reported to be involved closely in various physiological processes, including anti/prooxidant, anti-inflammation, cardio- and vascular-protection, neuroprotective activity, antimicrobial activity, as well as anti-carcinogenic properties [10]. A great deal of research has verified that RES worked via the following potential mechanisms: acts as the antioxidant or prooxidant being responsible for cytotoxic, or as the anti-proliferative and even pro-apoptotic molecular to induce apoptosis and/or cell cycle arrest against cancer [10, 11]. However, due to the low bioavailability and rapid metabolism rate [12], many researches were focused on the structure modification to further exploit its potential [13–15]. Up to now, structure modifications of RES were focused mainly on the olefinic bridge and/or the benzene ring parts [14]. Among these structural modification strategies, methylation had attracted a great deal of attention because of the simple synthesis and magical effect for the improvement of medicine potency [14, 16, 17]. Thus, to obtain anticancer drug molecules with excellent biological activity, we designed and synthesized resveratrol analogues **1**–**4** by methylation strategy in this work (Fig. 1). The cytotoxic activity of compounds and anticancer mechanism were further investigated subsequently. These results may provide an effective strategy for the design of anticancer reagents and the better understanding of anticancer mechanisms.

## Materials and methods

### Reagents

3-(4, 5-Dimethylthiazol-2-yl)-2,5-diphenyltetrazolium bromide (MTT), propidium iodide (PI), 4′,6-diamidino-2-phenylindole (DAPI), and 2′,7′-dichlorofluorescein diacetate (DCFH-DA) were from Sigma-Aldrich. Dulbecco’s Modified Eagle Medium (DMEM, High Glucose) and Minimum Essential Medium were purchased from Thermo Fisher Scientific. The normal human embryonic lung fibroblasts (MRC-5), human cervix adenocarcinoma cell (HeLa), and human breast cancer cell line (MCF-7) were purchased from cell culture center of Chinese Academy of Medical Sciences, the Institute of Basic Medical Sciences, authenticated by short tandem repeat (STR) profiling analysis (China Infrastructure of Cell Line Resource, Beijing, China). The human non-small cell lung cancer cell line (H1299) and immortalized normal human astrocyte cell line (UC2 provided by Southwestern Medical Center, University of Texas) were the gifts from Shi Cui-Juan (Tianjin Medical University) [18]. Detection of mycoplasma in all cell lines mentioned above was performed using polymerase chain reaction (PCR) methods [19]. Apoptosis detection kit was purchased from BD Biosciences. Antibodies against p53, p21, cyclin A1, cyclin A2, Bax, Bcl-2 and GAPDH were purchased from ABclonal.

### Methods

#### Synthesis of the compounds

The bis(tri-tert-butylphosphine)palladium(0) (30.6 mg, 0.06 mmol), cesium fluoride (334 mg, 2.2 mmol) and 4- bromophenol (2.5 mmol) containing different substituents were added into a round-bottom flask at room temperature, and the tributylvinyltin (0.3 mL, 1 mmol) and toluene (2.0 mL) were then added by syringe. The solution was heated and refluxed for 6 h under argon. The reaction liquid was cooled to room temperature, KF (500 mg) and ethyl acetate (5 mL) were added and stirred for 30 min, then extracted with ethyl acetate for three times. The organic phases were washed with a solution of saturated KF and water, dried over Na_2_SO_4_. The solvent was evaporated and the residue was purified by column chromatography (silica gel; petroleum/ethyl acetate 5/1) to provide compound **1**–**4**.

^1^H NMR and ^13^C NMR of compound **1**–**4** (**Additional file****1****: figure S1**.).

***Compound 1*** (*E*)-4,4′-(ethene-1,2-diyl)bis(3,5-dimethylphenol): m.p.: 224–226 °C; ^1^H NMR (400 MHz, acetone-*d*_6_): δ = 8.02 (s, 2H), 6.59 (s, 4H), 6.45 (s, 2H), 2.34 (s, 12H); ^13^C NMR (100 MHz, acetone-*d*_6_), δ = 156.6, 138.0, 132.1, 130.0, 115.6, 21.8.

***Compound 2*** (*E*)-4,4′-(ethene-1,2-diyl)bis(2,6-dimethylphenol): m.p.: 249–251 °C; ^1^H NMR (400 MHz, acetone-*d*_6_): δ = 7.29 (s, 2H), 7.14 (s, 4H), 6.88 (s, 2H), 2.24 (s, 12H); ^13^C NMR (100 MHz, acetone -*d*_6_), δ = 153.6, 130.5, 127.2, 126.5, 124.8, 16.6.

***Compound 3*** (*E*)-4,4′-(ethene-1,2-diyl)bis(2,5-dimethylphenol): m.p.: 299–300 °C; ^1^H NMR (400 MHz, acetone-*d*_6_): δ = 8.09 (s, 2H), 7.38 (s, 2H), 7.04 (s, 2H), 6.65 (s, 2H), 2.30 (s, 6H), 2.19 (s, 6H); ^**13**^**C NMR** (100 MHz, acetone -*d*_6_), δ = 153.6, 130.5, 127.2, 126.5, 124.8, 16.6.

***Compound 4*** (*E*)-4,4′-(ethene-1,2-diyl)bis(2,3-dimethylphenol): m.p.: 301–303 °C; ^1^H NMR (400 MHz, acetone-*d*_6_): δ = 8.15 (s, 2H), 7.28 (d, *J* = 8.4 Hz, 2H), 7.05 (s, 2H), 6.73 (d, *J* = 8.4 Hz, 2H), 2.28 (s, 6H), 2.17 (s, 6H); ^13^C NMR (100 MHz, acetone-*d*_6_), δ = 155.2, 136.1, 130.2, 127.7, 124.7, 123.3, 113.3, 15.8, 12.1

#### Cell culture

The cell lines were cultured in DMEM (MCF-7, HeLa, H1299, and UC2 involved) and MEM/NEAA (MRC-5) respectively, with 10% (v/v) fetal bovine serum (FBS), penicillin (100 units/mL), and streptomycin (100 units/mL), and kept at 37 °C in a 5% CO_2_ atmosphere. The cell lines were regularly subcultured.

DMSO, as the effective solvent, was used to dissolve the compounds at first. The concentration of DMSO in cell suspension was less than 0.1% (v/v).

#### Cell viability assay

The cell lines (MCF-7, HeLa, H1299, MRC-5 and UC2 involved) viability was evaluated by the MTT assay [20, 21]. The initial densities were 2.5 × 10^4^ cells/mL. After cell culture for 24 h, the culture medium was replaced, and the cells were incubated with target compounds at gradient concentrations (1 μM, 5 μM, 10 μM, 20 μM, 40 μM, 80 μM, four wells were used for each concentration, and repeat 3 times) for 72 h in 96-well flat microtiter plates. Subsequently, 100 μL culture medium containing 10% MTT was added to each well. Incubation for another 4 h at 37 °C in the dark, remove the culture medium, and add 100 μL DMSO in each cell. The absorbance was determined at 570 nm by a microplate reader (Thermo Fisher 1510).

#### Cell apoptosis analysis

Cell apoptosis assay was carried out by flow cytometry to detect labelled annexinV- fluorescein isothiocyanate/propidium iodide (Annexin V-FITC/PI). The initial densities of MCF-7 cells were 2 × 10^5^/mL in six-well plates for 24 h (1.5 × 10^5^/mL for 48 h). Then the culture medium was replaced by indicated concentrations of compound **1** (control, 5 μM, 10 μM and 20 μM) for another 24 h/48 h. After incubation, the cells were collected, washed twice with pre-cooling PBS, and stained with annexin V-FITC/PI for 15 min in the dark. The cells were evaluated by flow cytometry (BD FACS Calibur).

#### Nuclear staining with DAPI

The morphological changes of nucleus were observed by fluorescence microscopy with DAPI staining. Firstly, 8 × 10^4^/mL MCF-7 cells were planked in a 6-well plate with slide in each cell for 24 h. Later, the cells were treated with gradient concentration (control, 5 μM, 10 μM, 20 μM) of compound **1** for another 24 h. Then the treated cells were fixed with 4% paraformaldehyde overnight. DAPI staining for 10 min and the cells were determinated by fluorescence microscopy (Olympus BX41).

#### Cell cycle analysis

MCF-7 cells (2 × 10^5^/mL density) were seeded in six-well plates for 24 h. Discarding the culture medium, the MCF-7 cells were treated by different concentrations of compound **1** for another 24 h (control, 5 μM, 10 μM, 20 μM). Then the cells were collected and fixed with 70% ethanol at 4 °C overnight. Washed the cells with pre-cooling PBS later, and incubated with PI staining buffer for 30 min in the dark. Then the cell cycle distribution was analyzed by flow cytometry (BD FACSCalibur).

#### Measurement of ROS

DCFH-DA produces fluorescent signal after oxidation by ROS, which is used to reflect the intracellular level of ROS. MCF-7 cells were seeded in a 6-well plate for 24 h, and were treated with gradient concentration (control, 5 μM, 10 μM, 20 μM, 30 μM and 40 μM) of compound **1** for another 6 h or 9 h. Discarded the culture medium, collected and washed the MCF-7 cells, which were resuspended in PBS (3 μM DCFH-DA containing) for 0.5 h at 37 °C in the dark. Then washed the cells by PBS and detected the fluorescence intensity by flow cytometry (BD FACSCalibur) at once.

#### Western blot analysis

The lysates of treated MCF-7 cells with different concentration of compound **1** were extracted by RIPA Lysis Buffer containing PMSF (P0020, Solarbio, China) following the instructions. After centrifugation, the protein supernatants were collected, and the protein concentration was measured by BCA protein assay kit (R0020, Beyotime Institute of Biotechnology, China). Then the SDS-PAGE loading buffer (1:1 v/v) was added into the proteins sample, and boiled for 5 min. The extracted proteins were separated by SDS-PAGE and transferred to polyvinylidene difluoride (PVDF) membrane by a wet transfer method. After blocking with 5% nonfat milk in TBST for 1.5 h at room temperature, the PVDF membrane was incubated with primary antibodies (1:2000 against p53, p21, Bax, Bcl-2, Cyclin A1, Cyclin A2 and 1:10,000 against GAPDH) overnight at 4 °C, followed by the incubation with secondary antibody at room temperature for another 1.5 h. The immunoblots were visualized with an Ultra ECL kit (LK-U1421, MultiSciences Biotech Co., Ltd, China).

#### Statistical analysis

All experiments were repeated at least three times. The results were shown as mean ± SD and the differences were analyzed by ANOVA using SPSS 22.0.

## Results

### Synthesis of the compounds **1**–**4**

**Scheme 1:**
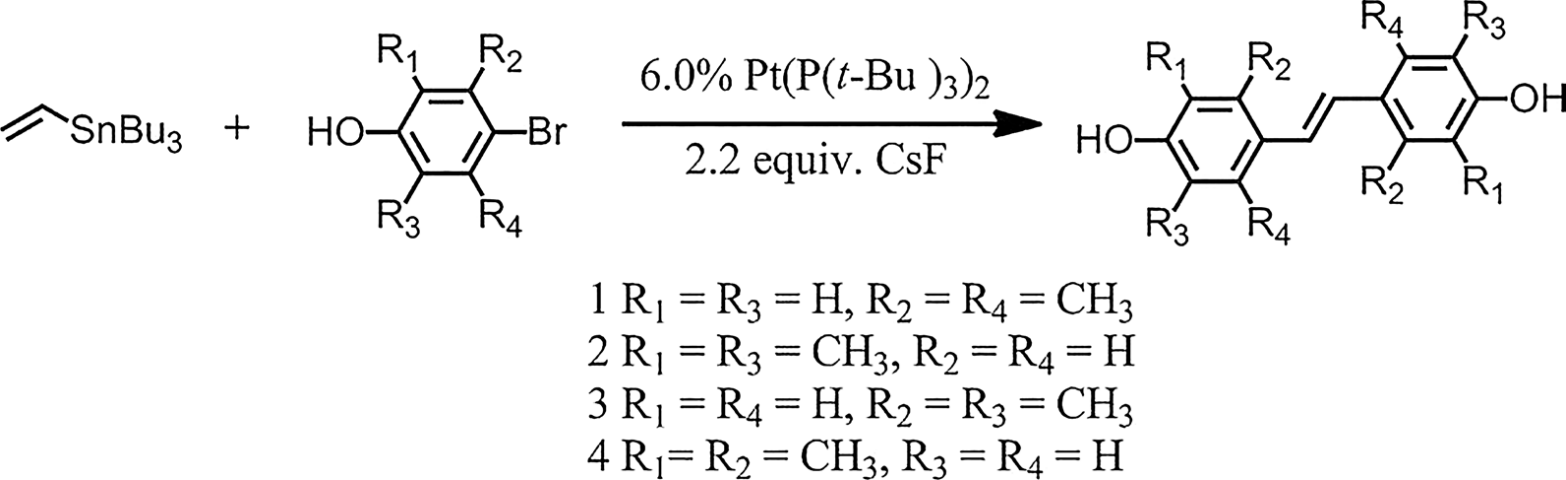
Synthesis of compound **1**–**4**

The compounds **1**–**4** (Fig. 1) were synthesized on basis of one step Heck and Stille series reaction (Scheme 1). Synthesis details and characterization obtained were described in "Methods" section.

### Cytotoxic activities of the resveratrol and compound **1**–**4**

Firstly, we investigated the anti-proliferative activities of resveratrol and compound **1**–**4** by MTT assay in various cell lines. As shown in Table 1, exhilaratingly, compound **1**–**4** exhibited more excellent cytotoxicity in MCF-7 cells compared to RES. Especially compound **1** possessed the obvious cytotoxicity in indicated tumor cells compared to that of other compounds. The introduction of four methyl groups on the aromatic rings can obviously improve the lipophilicity compared to RES (Table 1), which may benefit the drug molecules absorption and metabolism to promote their bioavailability. In addition, the four methyl groups in aromatic rings of ortho-position of the double bond (compound **1**) change the molecular plane angle into dihedral angle of molecular structure, which predisposes protein binding [14]. Therefore, methylation is an effective strategy for improving biological activities of hydroxystyrene. More importantly, except the broad-spectrum anti-cancer characteristics, the compound **1** exhibited the selectivity of anti-proliferative activity with about two times cytotoxicity in tumor cells (MCF-7, HeLa and H1299) than that in normal cells (MRC-5 and UC2). The excellent broad-spectrum anticancer activity and the good selectivity of anticancer activity between tumor cells and normal cells of compound **1** encouraged us to further research the mechanism of inhibitory effect on the tumor proliferation.

**Table 1:** Antiproliferative activities of resveratrol and its analogs compound **1**–**4** in cell lines

Compounds	IC_50_ (μM)^a^					Clog^*p*[b]^
	MCF-7	HeLa	H1299	MRC-5	UC2	
**1**	8.01 ± 0.35	8.36 ± 0.20	9.64 ± 0.48	18.67 ± 0.93	24.87 ± 2.62	5.496
**2**	13.16 ± 0.65	>80	45.28 ± 1.65	>80	68.73 ± 2.59	5.296
**3**	28.36 + 3.30	48.13 ± 0.95	28.87 ± 3.54	72.10 ± 3.18	46.97 ± 5.90	5.396
**4**	10.14 ± 1.59	9.85 ± 1.07	22.51 ± 2.84	24.02 ± 0.83	26.67 ± 0.83	5.296
RES	>80	>80	47.63 ± 1.62	51.3 ± 0.5	45.12 ± 4.65	2.833

^a^The results are presented as the mean ± SD for 4 replications in 3 separate trials after treatment with compounds for 72 h

^b^Calculated by Bio-Loom software [14]

### Induction of cell apoptosis in MCF-7 cells

**Fig. 2:**
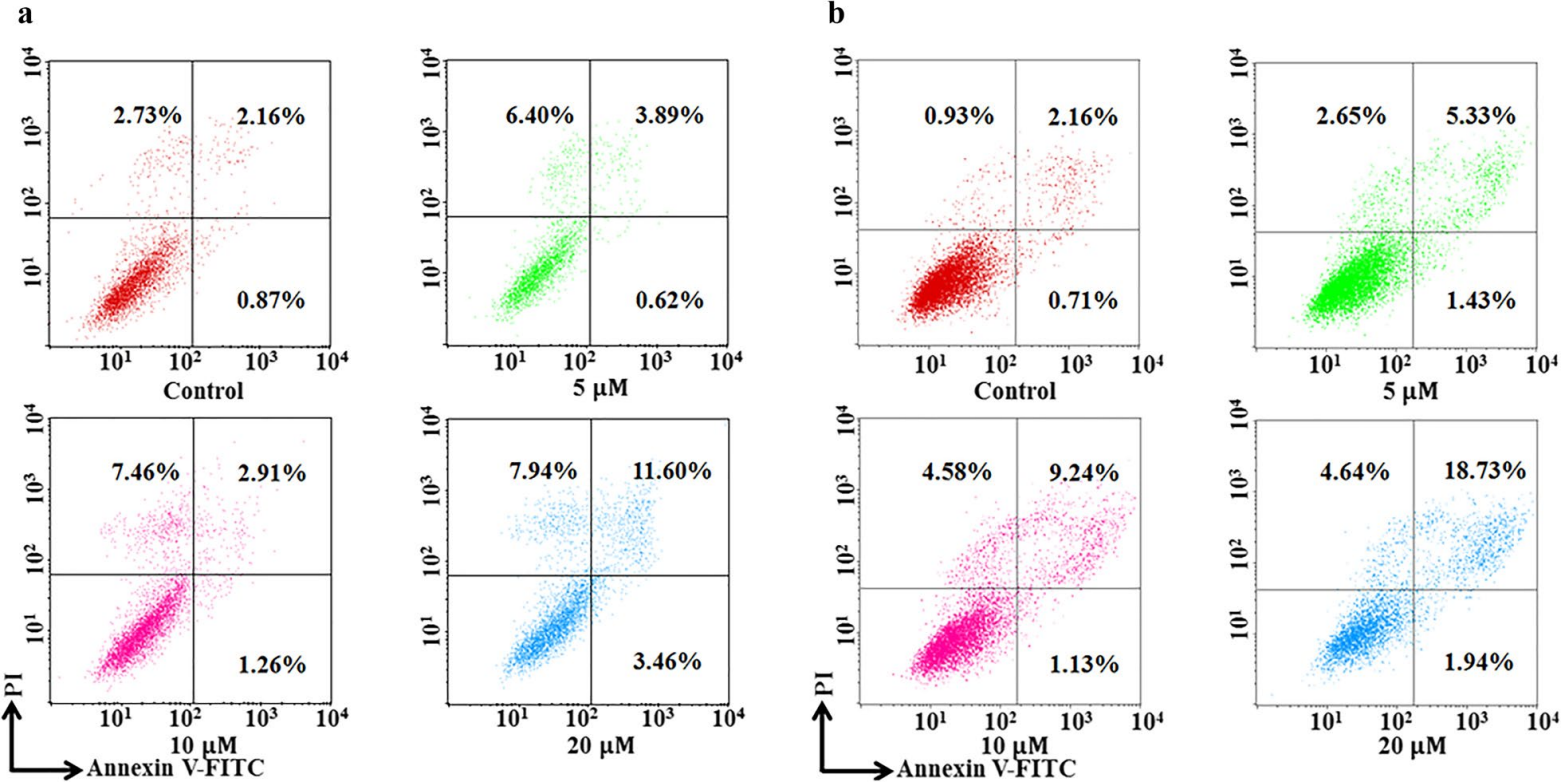
Compound **1** induced apoptosis in MCF-7 detected by flow cytometry. **a** MCF-7 cells were treated with gradient concentration (control, 5 μM, 10 μM, and 20 μM, respectively) for 24 h. **b** MCF-7 cells were treated with indicated concentration the same as **a** for 48 h. The percentage in each quadrant represents the proportion of early apoptotic cells, late apoptotic cells, necrotic and normal cells in the order quadrant 4**-**1**-**2**-**3

**Fig. 3:**
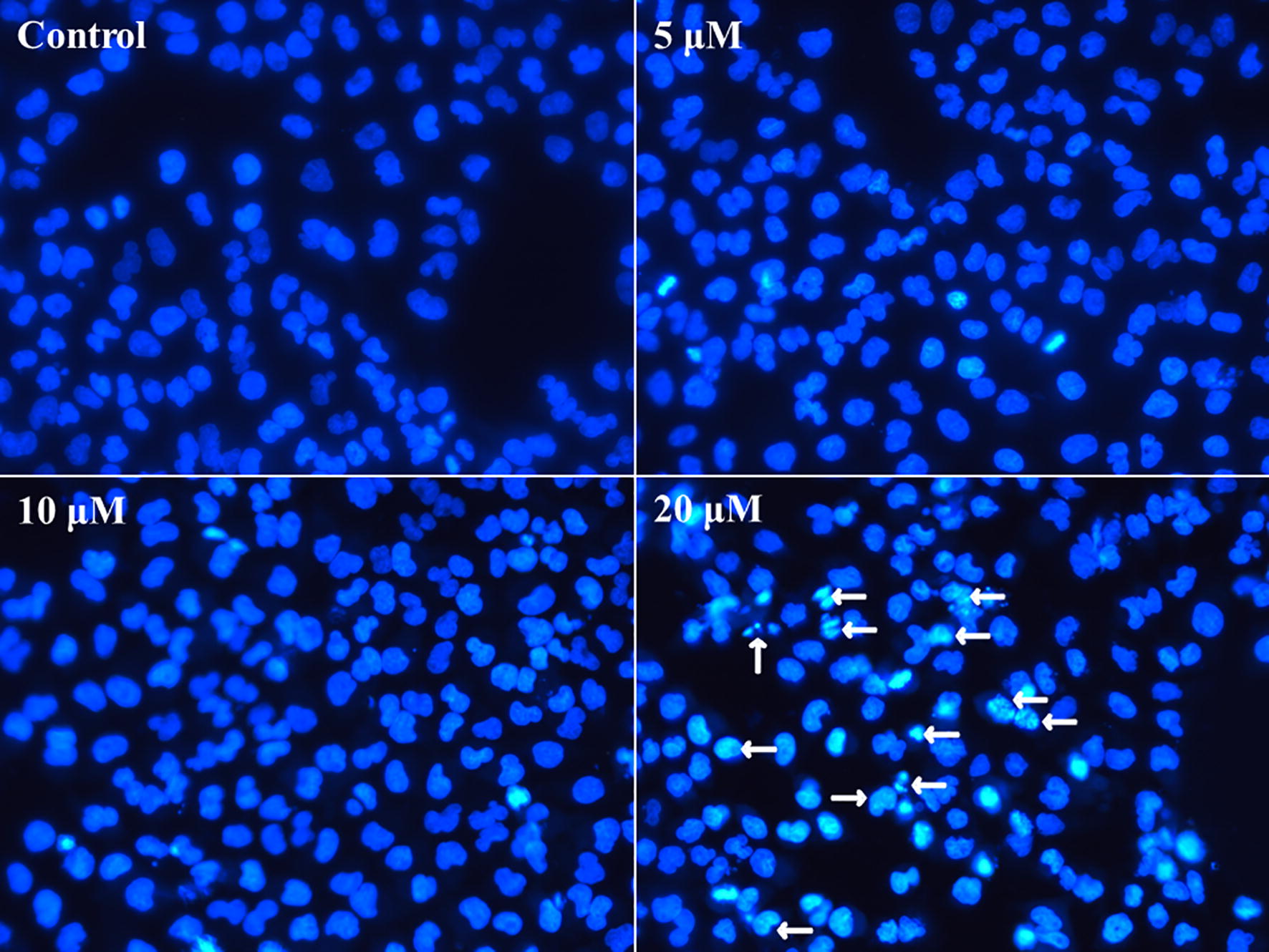
The nuclear chromatin condensation and the formation of apoptotic bodies with DAPI staining was demonstrated

Apoptosis, as programmed death, is an important way of cell death. Therefore, the effect of compound **1** on inducing apoptosis was detected by flow cytometry after Annexin V-FITC/PI double staining. The results showed that when the concentration of compound **1** increased to 20 μM for 24 h, the apoptosis was observed (Fig. 2a). As the extending of incubation time (48 h), the apoptotic effect showed concentration-dependent characteristics (Fig. 2b). On the other hand, apoptosis is a distinct characterised process with different morphological and biochemical features, so the DAPI staining was carried out to investigate the morphological changes of nucleus characteristic. After different concentrations of compound **1** treatment for 24 h, the MCF-7 cells presented chromatin condensation and nuclear fragmentation (arrow) at the concentration of 20 μM (Fig. 3), which further conformed apoptosis induction by compound **1** in MCF-7. Therefore, compound **1** is able to kill cancer cells by apoptosis manner.

### Induction of cell cycle arrest in MCF-7 cells

**Fig. 4:**
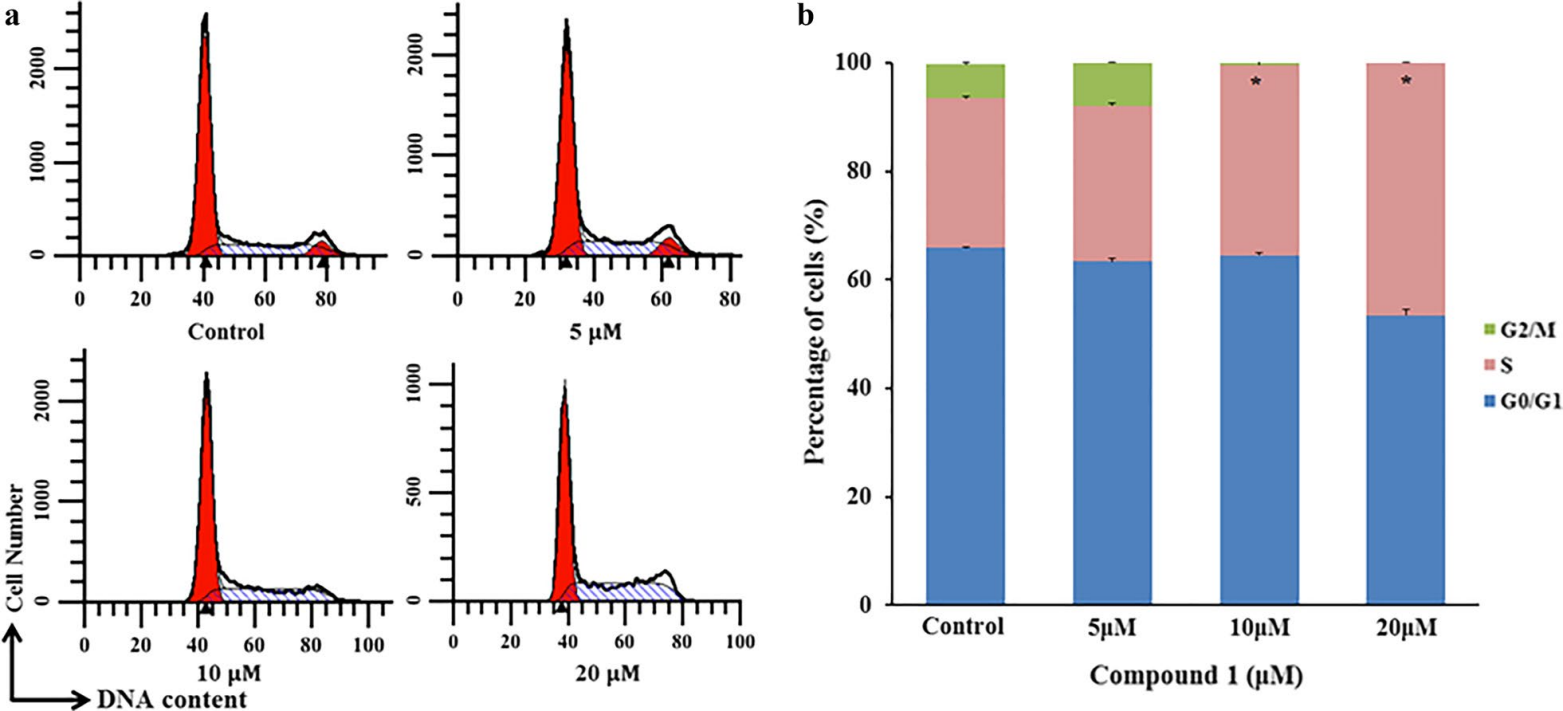
Compound **1** induced cell cycle arrest in S phase in MCF-7 detected by flow cytometry. **a** MCF-7 cells were treated with gradient concentration (control, 5 μM, 10 μM, and 20 μM, respectively) for 24 h. **b** The percentage in each column represents the proportion of cell cycle phases. The asterisks (*) marked in the graph represents the significant differences (*p *< 0.05) in different groups

Cell cycle is another factor of affecting the proliferation. In the following, the effect of cell cycle arrest was investigated. The result showed that compound **1** displayed the significant inhibitory effect on proliferation above 10 μM after incubation for 24 h, and the cells were arrested in S phase (Fig. 4). Meanwhile the effect showed concentration-dependent characteristics (Fig. 4b). Obviously, these results highlighted compound **1** inhibited tumor cell proliferation through binary pathways of cycle arrest and apoptosis which had been identified (Figs. 2 and 3).

### Effect of compound 1 induced ROS level in MCF-7 cells

**Fig. 5:**
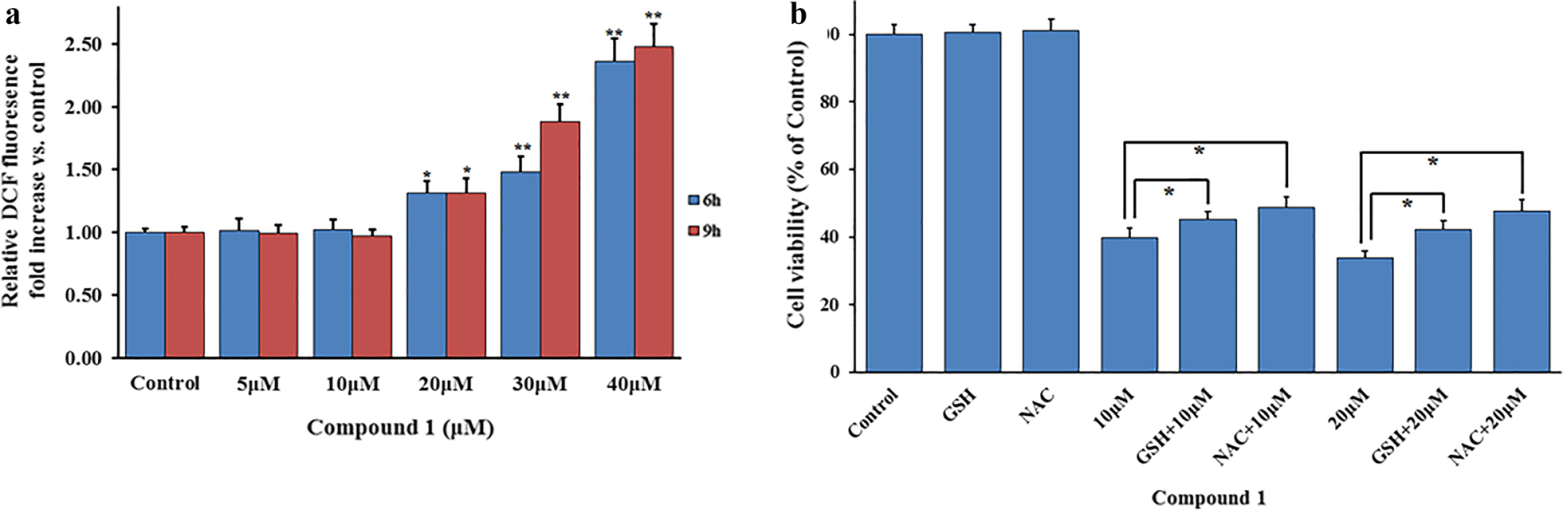
ROS induction by compound **1** may be the primary cause of apoptosis and cell cycle arrest. **a** ROS level significantly increased at high concentration (30 μM and 40 μM) after MCF-7 cells were treated with compound **1** for 6 h or 9 h. **b** The cell viability was reversed to some extent after a combined treatment of ROS scavengers (GSH 10 mM and NAC 10 mM) and compound **1** (10 μM and 20 μM, respectively) for 28 h. The asterisks (*) represents the significant differences (*p *< 0.05) in indicated groups

ROS, as the important second message, are responsible for regulating cellular activities. However, high levels of ROS may be toxic to cancer cells and suppress the tumor proliferation by induction of apoptosis and/or cell cycle arrest [8]. Therefore, the ROS level in MCF-7 cells was detected by flow cytometry. As shown in Fig. 5a, the ROS level expressed a slight increase at 20 μM after compound **1** treatment for 6 h and 9 h (Fig. 5a), but showed a significant rise with the increase of the concentration. Interestingly, when the ROS scavengers, including GSH and NAC, were added to the cells 1 h earlier than compound **1**, the cell survival rate was reversed to some extent (Fig. 5b). Thus, compound **1** may increase intracellular ROS level, which may further induce the apoptosis and cell cycle arrest signal pathway to inhibit cellular activity.

### Effect of compound 1 on the expression of *p53, p21, Bax* and *Bcl*-*2* in MCF-7 Cells

**Fig. 6:**
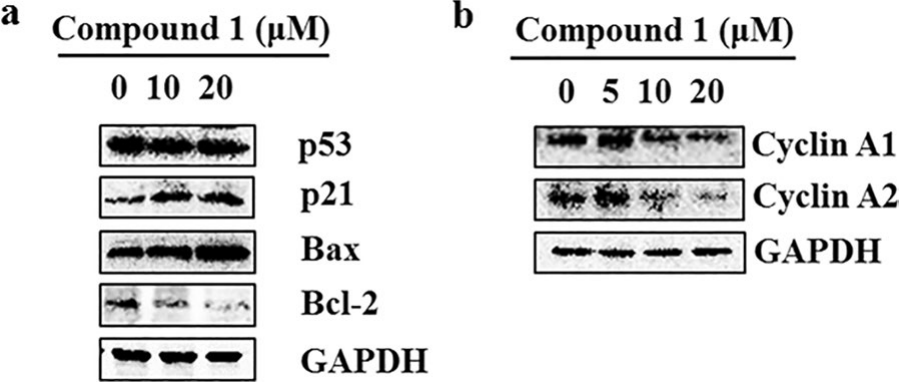
The expression of apoptosis-related proteins and cycle-regulatory proteins of S phase was evaluated. **a** Compound **1** promoted apoptosis by affecting the expression of apoptosis-associated proteins. **b** Compound **1** induced cell cycle arrest in S phase by down-regulating the expression of cycling A1 and cycling A2

Whereafter, we further investigated the expression levels of apoptosis-related proteins to explore the anticancer mechanism of compound **1**. Hence, the expression levels of apoptosis-associated proteins including p53, p21, Bax and Bcl-2 were tested after treatment for 24 h with compound **1** in MCF-7 cells. Exhilaratingly, the results showed that compound **1** could induce up-regulation of pro-apoptotic protein Bax and down-regulation of anti-apoptotic protein Bcl-2, which increased the Bax/Bcl-2 ratio (Fig. 6a) compared to untreated MCF-7 cells. More importantly, as the tumor suppressor and the upstream regulatory protein of apoptosis pathway, p21 showed a marked increase (Fig. 6a). However, there was only little difference in p53 between treated and untreated groups.

### Effect of compound 1 on the expression of *cyclin A1* and *cyclin A2* in MCF-7 Cells

Based on the effect of compound **1** on the cell cycle progression, cyclin A1 and cyclin A2 expression related to S phase were tested by western blot analysis. As shown in Fig. 6b, compared to untreated cells, there was a lower expression of cycling A1 and cycling A2 in MCF-7 with compound **1** treated at concentration of 10 μM and 20 μM for 24 h, which provided an explanation of blocking cell cycle in S phase. Therefore, compound **1** blocked cell cycle in S phase with the down-expression of cycling A1 and cycling A2. Obviously, compound **1** inhibits cancer cells proliferation through the modulation of the expression of apoptosis-related proteins and cyclin proteins.

## Discussion

Carcinogenesis is a complex and heterogeneous process, with typical characteristic, namely, uncontrolled cells division. Obviously, multiple mechanisms inhibiting cell proliferation, which were especially associated with apoptotic signal pathway and cell cycle regulation, become the preferred option of anticancer targets [3, 22, 23], and the therapeutic strategies targeting intracellular ROS levels, which were influenced by the natural products and their structural analogues act as prooxidants, were the research hotspots [8, 24]. In this study, tetramethylated hydroxystyrene of resveratrol analogues, namely, compound **1**–**4**, were designed and synthesized based on the resveratrol skeleton units. Although the synthesis of compounds have been previously reported [25–27], the inhibitory effect on tumor cell growth and the anticancer mechanisms are not studied. Therefore,we firstly investigated the cytotoxic activities of the compounds. Encouragingly, compound **1** showed a higher anti-proliferative activity and selectivity of anticancer action compared with that of resveratrol, and showed the broad-spectrum anti-cancer characteristics compared to other synthesized compounds (Table 1). According to the subsequent studies, compound **1** expressed markedly inhibitory effect in the MCF-7 cells by both cell cycle arrest and pro-apoptosis activity. This result showed that the introduction of four methyl groups in ortho sites of aromatic rings significantly improved its cytotoxicity. For that reason, the distortion of distyrene space structure with introduction of methyl group may bond the protein more closely.

As we known, apoptosis is a key factor in leading to the cell death, which may be induced by various stresses such as therapeutic agents through induction ROS by triggering oxidative stress [28, 29]. In this study, ROS levels were markedly elevated at a higher concentration, and the cell viability was also enhanced after ROS scavenger combined with compound **1** treatment (Fig. 5). Obviously, these results supported compound **1** upregulated the ROS levels to mediate the cell apoptosis and inhibit the proliferation of cancer cells. The main mechanisms may be referred to the imbalance of pro-apoptotic and anti-apoptotic proteins or/and the activation of caspase pathway [5]. Interestingly, our studies showed that the pro-apoptotic protein Bax (up-regulation) and anti-apoptotic protein Bcl-2 (down-regulation) promoted a shift in the Bax/Bcl-2 ratio toward apoptosis (Fig. 6a). Meanwhile, as a master apoptosis modulator in response to the stimuli and the upstream regulation protein of apoptosis pathway by both p53-dependent and p53-independent pathways [30], p21 showed an obvious up-regulation in a dosage dependent manner after compound **1** treatment. However, the expression of p53, as a tumor suppressor and a key regulator in apoptosis induction [31], was not obvious change in the present study, which indicated that the increased expression of p21 caused by compound **1** in p53-independent may be an important reason for the apoptosis of tumor cells. However, due to the caspase-3 activity deficiency in MCF-7 cells [32], compound **1** didn’t induce caspase-3 activity at high concentrations in this study (result not show). Therefore, the realization of apoptosis induced by compound **1** mainly depends on p21 rather than upstream protein p53.

On the other hand, cycle arrest is another important manner for affecting the cell activities. The cell cycle traverses the four distinct phases, including G1, S, G2, and finally M phase, which are guarded by G1/S, intra-S, and G2/M checkpoints in response to DNA damage or cellular perturbations [33]. Numerous researches suggested that ROS, as key signaling intermediates, could damage the DNA and disturb the cell cycle progression by phosphorylation or ubiquitination of cell cycle regulatory proteins including cyclins and cyclin-dependent kinase (CDKs). [29, 34, 35]. In this study, the cell cycle analysis result verified that the MCF-7 cells were arrested in S phase after compound **1** treatment (Fig. 4), which indicated that compound **1** might inhibit the proliferation activity underlying the ROS- induced DNA damage, and then arrest cell cycle in S phase. In addition, S phase progression is regulated by the cycling A/CDK2 complex or complexes [23]. Obviously, down-regulated expression of cycling A1 and cycling A2 in MCF-7 cells after compound **1** treatment in the study provided a proper explanation of blocking cell cycle in S phase (Fig. 6b). Moreover, p21, as a vital cell cycle inhibitor, serves a role by inhibiting cyclin-CDK complexes [36]. The expression of p21 showed obvious upregulation in a dosage dependent manner accompany with ROS levels after compound **1** treatment, which indicated compound **1** might induce cell cycle arrest by upregulation p21 interacting with cycling A/CDK2 complex or complexes which are required for S phase progression to inhibit cellular proliferation. p53, as a key regulator in cell cycle regulation, plays an important in regulating the cell proliferation [5, 31]. However, the expression level of p53 was not obvious change in the present study, which suggested the cell cycle arrest might be p53-independent and chiefly regulated by induction of DNA damage via ROS in a p21-dependent manner, which reached a similar conclusion just like apoptosis study. Some mechanism research has showed that resveratrol and its analogues inhibit the proliferation of tumor cells by binding quinone reductase 2, glutathione sulfotransferase and estrogen receptor-β, inhibitor of kappa B kinase (IKK) [37, 38]. However, in this work, the possible target protein of is still uncertain, which is being studied in our lab.

## Conclusion

The study indicated that the tetramethylated hydroxy distyrene of resveratrol analogue, compound **1** showed significant and selective anticancer activities by both apoptosis induction and cell cycle arrest in S phase involving p21-dependant pathway in a prooxidant manner. However, the exact mechanism and potential therapeutic application of compound **1** is being continuing studied in this team.

## Supplementary information

**Additional file 1: Figure S1.**^1^H NMR and ^13^C NMR of compound **1**–**4**.

## Data Availability

Data supporting our findings is contained within the manuscript. Additional data will be shared upon request to the corresponding author.

## Abbreviations

ROS: Reactive oxygen species
RES: Resveratrol (3,4′,5,-trihydroxy-trans-stilbene)
MTT: 3-(4, 5-Dimethylthiazol-2-yl)-2,5-diphenyltetrazolium bromide
PI: Propidium iodide
DAPI: 4′,6-Diamidino-2-phenylindole
DCFH-DA: 2′,7′-Dichlorofluorescein diacetate
DMEM: Dulbecco’s Modified Eagle Medium
FBS: Fetal bovine serum
MRC-5: Normal human embryonic lung fibroblasts
HeLa: Human cervix adenocarcinoma cell
MCF-7: Human breast cancer cell line
STR: Short tandem repeat
H1299: Human non-small cell lung cancer cell line
UC2: Immortalized normal human astrocyte cell line
PCR: Polymerase chain reaction
PVDF: Polyvinylidene difluoride
