# Supplementary material for: Finding an efficient tetramethylated hydroxydiethylene of resveratrol analogue for potential anticancer agent
Source: BMC Chem. 2020 Feb 18;14(1):13. doi: 10.1186/s13065-020-00667-5 (PMC7027093; doi:10.1186/s13065-020-00667-5)

**SUPPORTING INFORMATION**

Finding an efficient tetramethylated hydroxydiethylene of resveratrol analogue for potential anticancer agent

**Zhen-Hui Xin^1#^, Ya-Li Meng^1#^, Wen-Jing Jiang^1^, Ya-Peng Li^2^, Li-Ping Ge^1^, Cun-Hui Zhang ^1^, Lian-Na Liu^1^, Yan-Fei Kang^1^,***

^1^ Hebei Key Laboratory of Quality & Safety Analysis-Testing for Agro-Products and Food and College of Laboratory Medicine, Hebei North University, 11 Diamond Street South, Zhangjiakou, 075000, Hebei Province, People’s Republic of China

^2^ Zhangbei Hospital, Guangchang Alley, Garden Street, Zhangbei Country, Zhangjiakou, 076450, Hebei Province, People’s Republic of China.


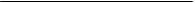


*For correspondence

Contact Information: +8618931319293; Fax: +86-313-4029275.

E-mail: kangyanfei172@163.com (Y.-F. Kang)

^#^Zhen-Hui Xin and Ya-Li Meng contributed equally to this work.

**1. Figures S1 ^1^H NMR and** **^13^C NMR of** **compound 1-4**


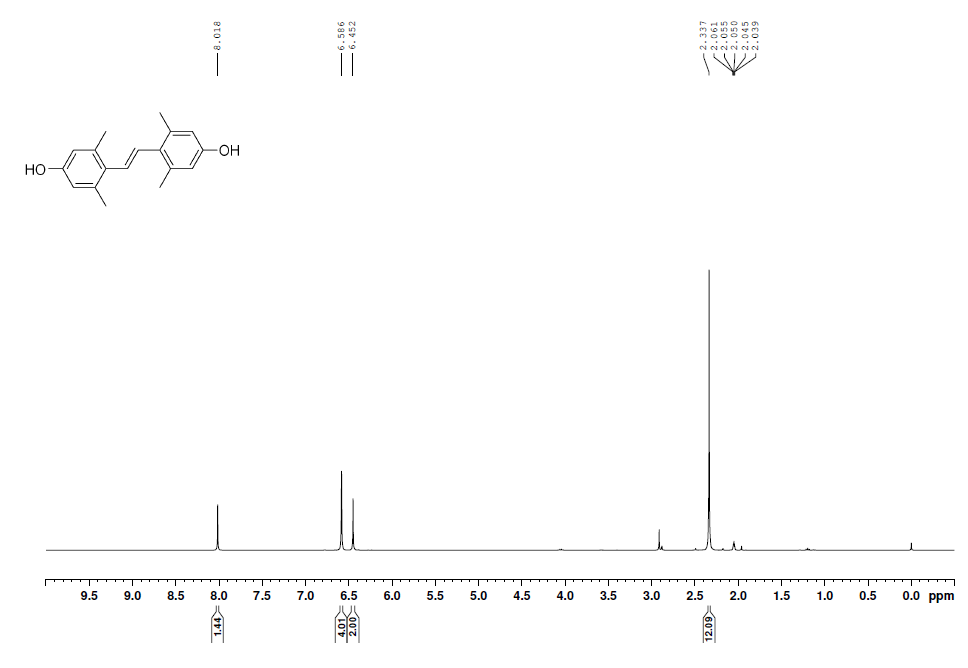


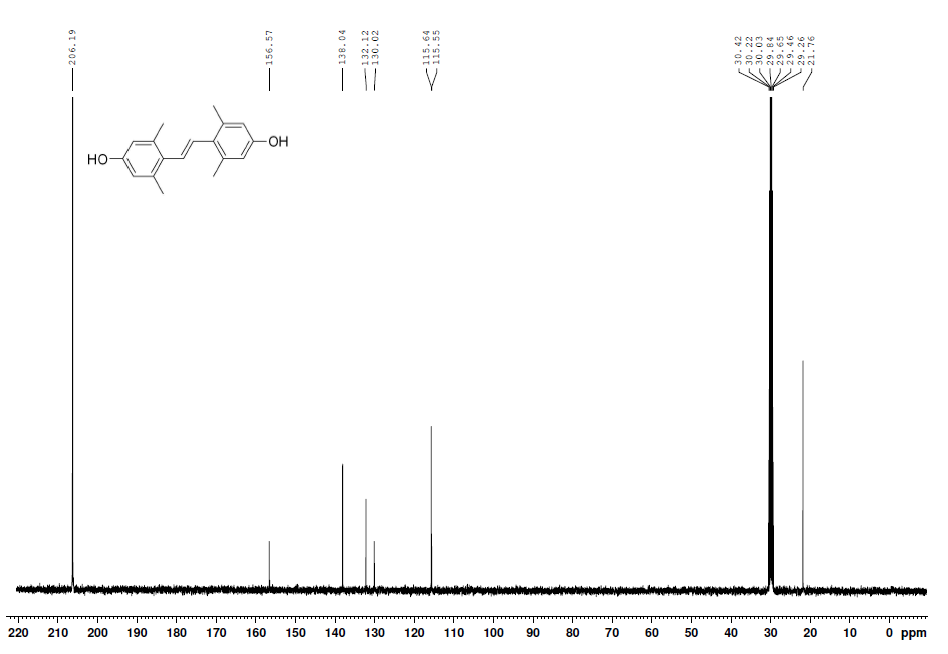


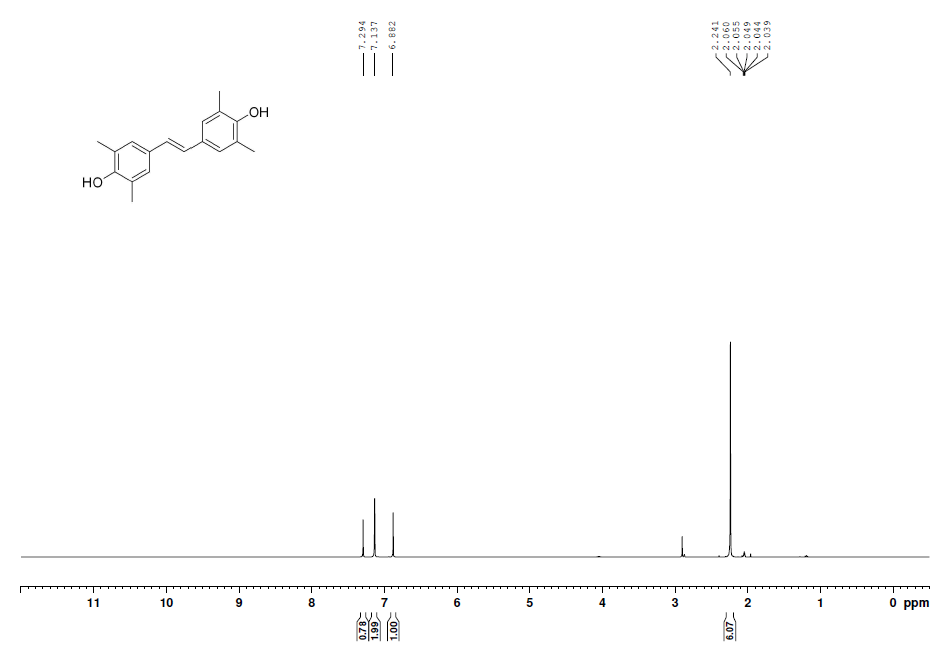


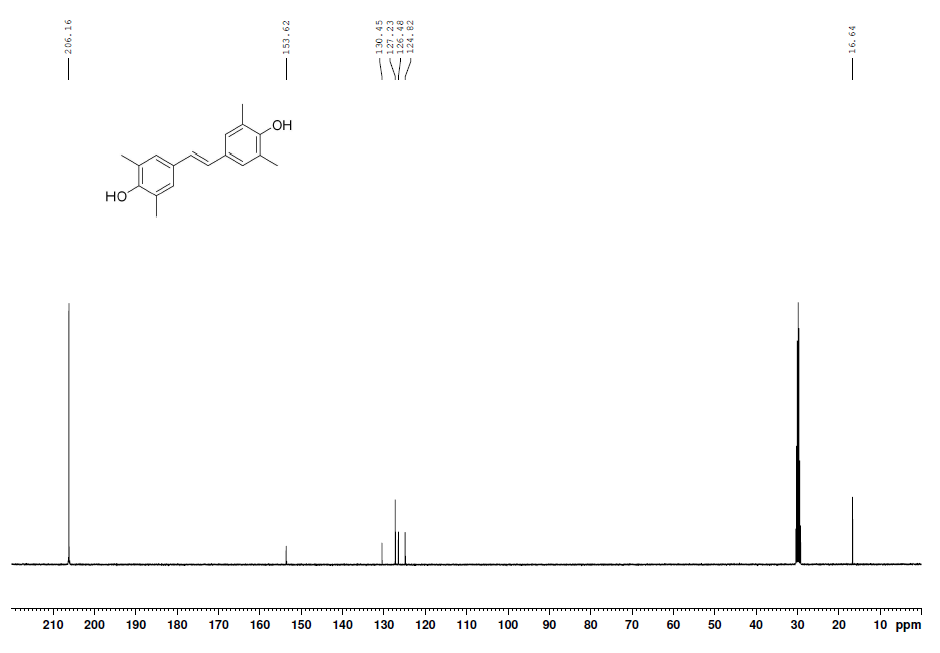


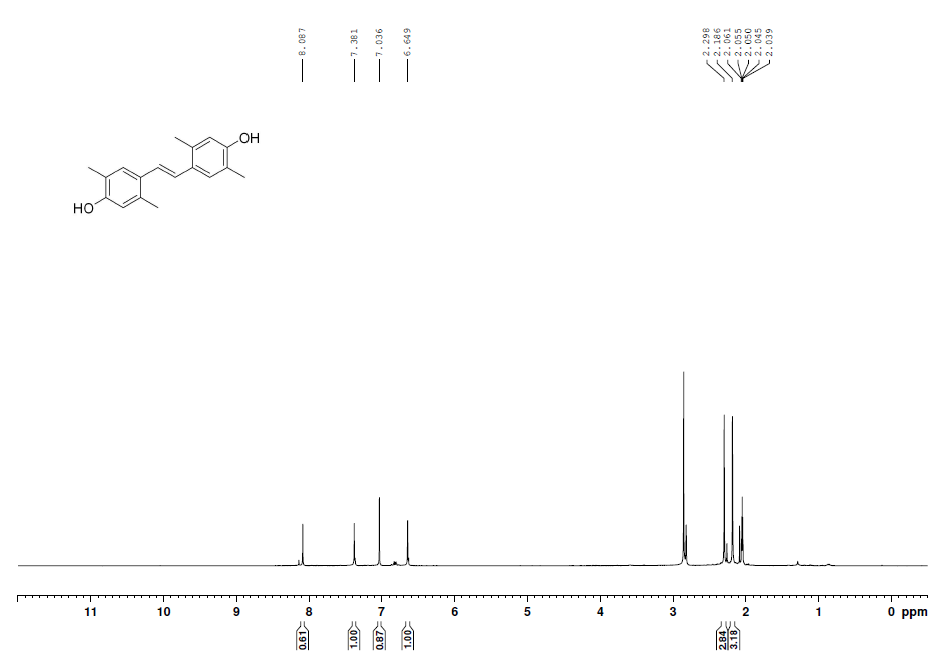


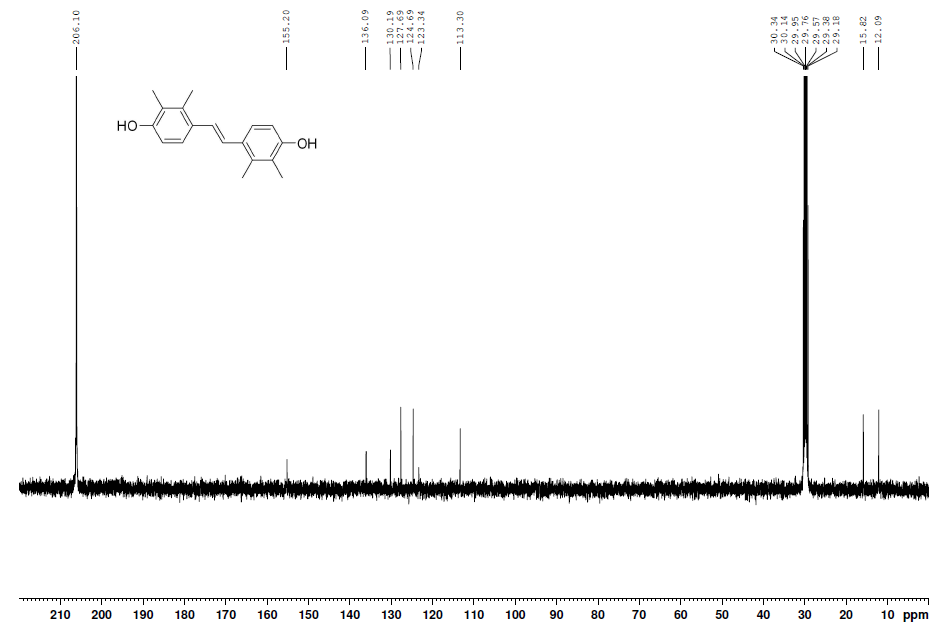


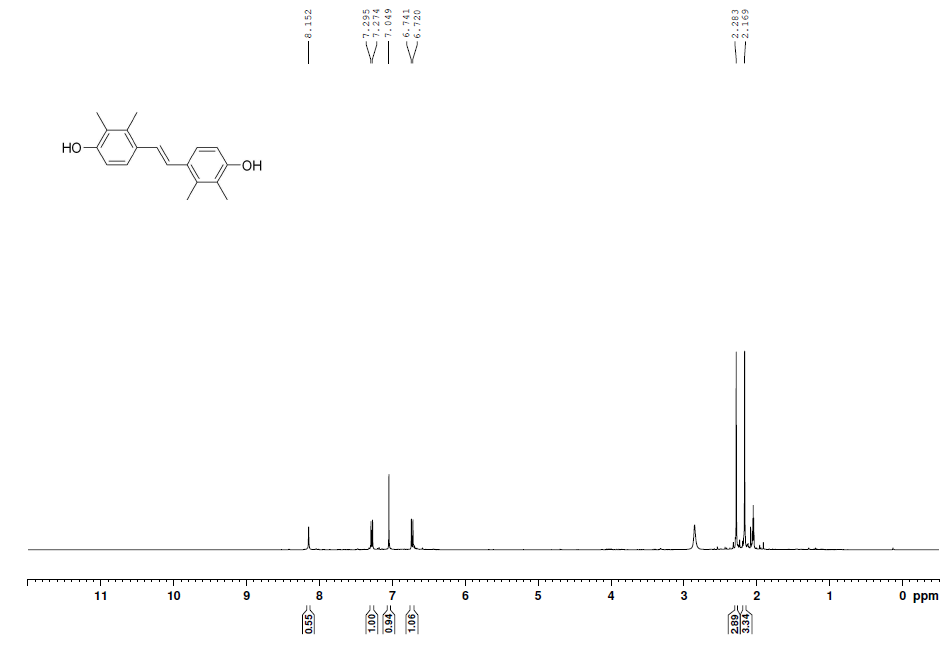


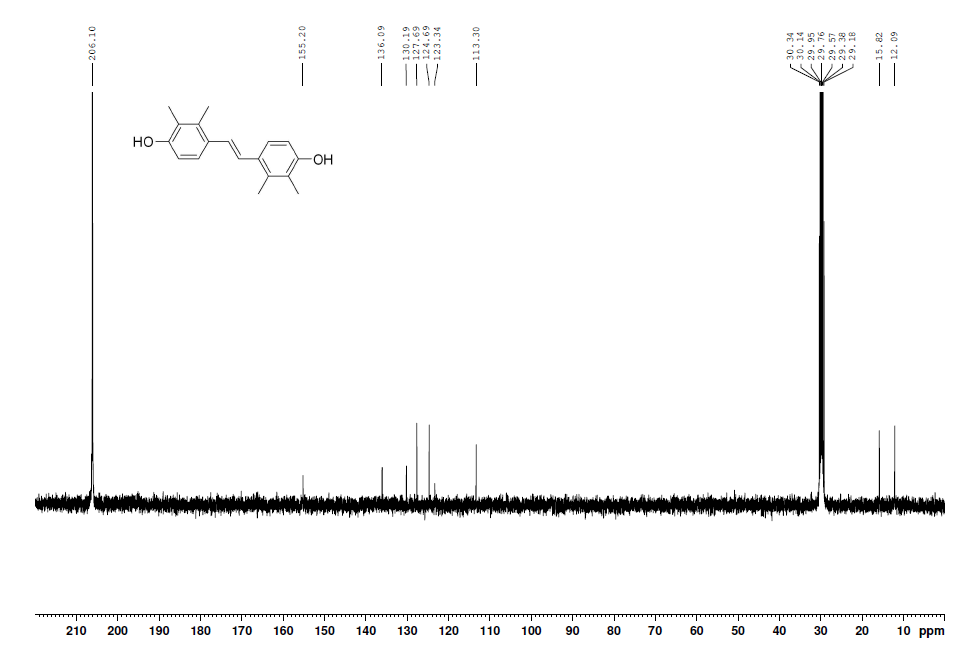

Supplement: Supplementary file 1 — Additional file 1: Figure S1.1H NMR and 13C NMR of compound 1–4. [file 13065_2020_667_MOESM1_ESM.doc]
